# Human papillomavirus infection in head and neck cancer: The role of the secretory leukocyte protease inhibitor

**DOI:** 10.3892/or.2013.2327

**Published:** 2013-03-05

**Authors:** MARKUS HOFFMANN, ELGAR S. QUABIUS, SILKE TRIBIUS, LENA HEBEBRAND, TIBOR GÖRÖGH, GORDANA HALEC, TOMAS KAHN, JÜRGEN HEDDERICH, CHRISTOPH RÖCKEN, JOCHEN HAAG, TIM WATERBOER, MARKUS SCHMITT, ANNA R. GIULIANO, W. MARTIN KAST

**Affiliations:** 1 Department of Otorhinolaryngology, Head and Neck Surgery, Christian-Albrechts University Kiel, D-24105 Kiel; 2 Institute of Immunology, Christian-Albrechts University Kiel, D-24105 Kiel; 3 Department of Radiation Oncology, University Medical Center Hamburg-Eppendorf, D-20246 Hamburg; 4 German Cancer Research Center (DKFZ), Infection and Cancer Program (F020), D-69120 Heidelberg; 5 Expert Team Life Sciences, Deutsche Bank AG, D-60311 Frankfurt; 6 Institute for Medical Informatics and Statistics, University Hospital of Schleswig-Holstein, Campus Kiel, D-24015 Kiel; 7 Institute for Pathology, Christian-Albrechts-University Kiel, D-24105 Kiel, Germany; 8 Center for Infection and Cancer Research, and the Department of Cancer Epidemiology, H. Lee Moffitt Cancer Center, Tampa, FL 33612; 9 Departments of Molecular Microbiology and Immunology, and Obstetrics and Gynecology, Norris Comprehensive Cancer Center, University of Southern California, Los Angeles, CA 90033, USA

**Keywords:** head and neck squamous cell carcinoma, human papillo- mavirus, secretory leukocyte protease inhibitor, p16

## Abstract

We previously showed that secretory leukocyte protease inhibitor (SLPI) gene and protein expression is significantly lower in metastatic versus non-metastatic head and neck squamous cell carcinoma (HNSCC). However, we did not assess the human papillomavirus (HPV) status of these cases. Since SLPI plays a role in HIV and herpes simplex virus (HSV) infections, we hypothesized that SLPI may be involved in HPV-infected HNSCC. In HNSCC tissue (n=54), HPV DNA was determined and correlated with SLPI expression. Additionally, to investigate a possible role of smoking on SLPI expression in clinically normal mucosa, 19 patients treated for non-malignant diseases (non-HNSCC) were analyzed for SLPI expression and correlated with smoking habits. In HNSCC patients, SLPI expression showed a significant inverse correlation with HPV status. In patients with moderate/strong SLPI expression (n=19), 10.5% were HPV-positive. By contrast, patients with absent/weak SLPI expression (n=35), 45.7% were HPV-positive. Low SLPI expression was correlated with metastasis (P=0.003) independent of HPV status. HPV-positivity was clearly associated with lymph node status (81.3% N1-3 cases). In smoking non-HNSCC patients (n=7), 42.9% showed absent/weak and 57.1% moderate/strong SLPI staining. In non-smoking non-HNSCC patients (n=10) 83.3% showed absent/weak and 16.7% moderate/strong SLPI expression. For the first time, a correlation between SLPI downregulation and HPV infection was demonstrated, suggesting that high levels of SLPI, possibly induced by environmental factors such as tobacco smoking, correlate with protective effects against HPV infection. SLPI may be a potential biomarker identifying head and neck cancer patients not at risk of developing metastases (SLPI-positive), and those at risk to be infected by HPV (SLPI-negative) and likely to develop metastases.

## Introduction

We previously demonstrated that secretory leukocyte protease inhibitor (SLPI) is significantly associated with head and neck squamous cell carcinoma (HNSCC) (1). Although we did not assess human papillomavirus (HPV) status, the viral etiological agent in a subset of HNSCC (2–5), we, and others, repeatedly showed a correlation between HPV infection and metastases of these tumors (6–8). Additionally, SLPI has been associated with other viral infections and it has been demonstrated that the prevalence of oral HIV is reduced in cases of elevated SLPI expression (reviewed in ref. 9). By contrast, herpes simplex virus (HSV) downregulates SLPI levels in a cell culture model (10). Therefore, we evaluated the association between HPV status and SLPI expression in a series of tumors using multiple markers of HPV infection across multiple anatomical sites within the head and neck and correlated this with occurrence of metastases.

The SLPI protein, also known as antileukoprotease, is a 11.7-kDa (107 amino acids) non-glycosylated kazal-type serine protease inhibitor of neutrophil elastase, cathepsin G, chymotrypsin and trypsin (11). It is produced by different cell types including breast, lung, endometrium, ovary, salivary glands and by various host inflammatory and immune cells such as macrophages, neutrophils and B lymphocytes (12–14). In particular, the role of SLPI as a potent inhibitor of neutrophil elastase appears to be an important factor resulting in the protection of the mucosa and skin against proteolysis (13,15).

In HNSCC, two possible types of carcinogenesis are discussed: i) an HPV-driven carcinogenesis, leading to biologically more aggressive tumors including early metastatic spread into the locoregional lymph nodes, but with comparatively better survival rates of the patients (8,16); and ii) a tobacco/alcohol-driven carcinogenesis, leading to delayed onset of disease with a time lapse of approximately 10 years (17). However, recent data suggest a synergism between HPV and smoking (16).

Moreover, a recent report showed a positive correlation between cigarette smoke and SLPI-expression in a rat model (18). This finding motivated us to further study SLPI expression in healthy mucosal tissue of non-HNSCC patients and to correlate SLPI expression levels with smoking habits.

## Materials and methods

### Patients, tissue specimens, DNA and RNA extraction

From 2004 to 2009, tissue samples of histopathologically confirmed HNSCC were obtained from 54 patients (42 male and 12 female; range, 43–76 years; median, 58 years) during surgery at the Department of Otorhinolaryngology, Head and Neck Surgery at the Christian-Albrechts-University Kiel, Germany. All samples were obtained following informed consent approved by the local Ethics Committee (D 438/10). Tissue samples were treated as follows: one section of the tumor tissue was snap-frozen in liquid nitrogen and stored at −80°C for further analysis. The remaining section of each tumor was processed for routine histopathology. DNA was extracted from 25 mg frozen tissue samples, as previously described (20,19). Total RNA was extracted using peqGOLD TriFast reagent (PeqLab, Erlangen, Germany) according to the manufacturer’s protocol.

The anatomical location of the primary tumors was: tonsils [(n=20); palatine tonsil (n=14), lingual tonsil (n=6)], larynx (n=22), tongue (n=8), and soft palate (n=4). SCC of the tongue (anatomical localization of the oral cavity) and SCC of the soft palate (per definition anatomical localization of the oropharynx) were investigated in one subgroup (n=12) since the biological behavior of soft palate SCC is more comparable to oral cavity SCC than to tonsillar SCC (per definition anatomical localization of the oropharynx). Additional clinicopathological characteristics and tobacco smoking habits are presented in Table I.

We also obtained tissue samples from clinically normal mucosa of the aerodigestive tract from 19 patients (12 male and 7 female; range, 2–63 years; median, 43 years) who were treated for non-malignant diseases at our Department. Additional clinicopathological characteristics and tobacco smoking habits are presented in Table II.

### Detection of HPV DNA

Two different PCR-based detection methods for HPV DNA were applied to increase precision in validity and reliability of HPV DNA diagnostics: multiplex HPV genotyping (MPG) assay and HPV^Type^ 3.5 LCD-Array, as previously described (20–22; Table I).

### Detection of HPV E6*I mRNA in fresh frozen tissue

In cases with RNA available for analysis (n=37), total RNA was subjected to ultra-short type-specific RT-PCR assays which were developed for 12 HR- and 8 pHR-HPV to amplify ~65 bp of the E6*I mRNA (23). RNA integrity was assessed by co-amplification of 81 bp of ubiquitin C transcripts. PCR products were detected by Luminex hybridization. The cut-off value (5 net MFI) to define HPV E6*I mRNA-positivity was applied as previously described (23).

### Immunohistochemistry for SLPI

Paraffin-embedded tissue specimens were cut into 5-μm sections, and stained for SLPI expression as previously described (1).

The slides were reviewed by two experienced members of the Department of Otorhinolaryngology, Head and Neck Surgery, Kiel, and by an experienced pathologist. To assess SLPI protein levels, 300 cells in at least five areas were analyzed. The mean percentage of positive tumor cells was determined and cases were assigned to one of the following categories: (−) <5%; (+) 5–30%; (++) 31–75%; and (+++) >75%. Cases with a score of (−) were considered negative, cases with scores of (+, ++, +++) positive.

### Algorithm for defining HPV-positivity

In the present study, specimens were classified as HPV DNA-positive only if the HPV DNA was detected by the HPV^Type^ 3.5 LCD-Array and/or in the presence of high viral loads by BSGP5+/6+-PCR-MPG assay. HPV infections were classified as active if HPV E6*I mRNA was detected. When RNA was not available, HPV DNA-positive cases with p16^INK4A^ overexpression were classified as active.

### Statistical analyses

Fisher’s exact test was performed to test for differences between groups, using the SAS 9.2 software. All tests were performed two-sided, and P-values <0.05 were considered to indicate statistically significant differences.

## Results

### Clinicopathological characteristics and smoking habits of HNSCC patients

The clinicopathological assessment, including the HPV detection, HPV E6*I mRNA analysis, SLPI immunohistochemistry and smoking habits are summarized in Table I. The inverse correlation between smoking habits and HPV status is statistically significant (P=0.02), whereas the correlation between SLPI expression and smoking habits is not. However, it is evident that all cases with moderate/strong SLPI expression can be assigned to heavy smokers and that most of the non-smokers showed absent/weak SLPI expression.

### HPV DNA analysis

HPV DNA was detected in 33.3% of the analyzed cases. HPV16 and HPV18 were the only infecting HPV types in 88.9 and 11.1% of all cases, respectively. From the two HPV18-positive cases, 1 was HPV18 DNA alone and the other was a double infection with HPV16.

Anatomical distribution of the tumor and their HPV levels was: from 20 tonsillar carcinomas, 60% tested positive for HPV DNA, including the HPV18 case and the case with double infection (both palatine tonsil). From the 22 specimens derived from laryngeal carcinomas, 18.1% were infected with HPV16. From the 12 carcinomas of the tongue and the soft palate, 16.7% were HPV16-positive. The prevalence was significantly higher in the tonsillar tumors (P=0.009).

### Expression of E6*I mRNA and activity status of HPV infection

Cases with available RNA (n=37) were subjected to HPV E6*I mRNA analysis. Signals for HPV RNA were detected in 17 cases, of which 14 could be assigned to HPV DNA-positive cases. The remaining 3 cases were classified as HPV-negative due to lack of HPV DNA-positivity according to the applied algorithm. However, considering HPV RNA expression as more important for an active HPV infection than existence of HPV DNA, these 3 cases would need to be classified as cases with active HPV infection. Only 1 out of the 17 HPV DNA positive cases with available RNA did not show detectable HPV mRNA. This case was classified as inactive infection, whereas the 14 HPV DNA positive cases with detectable levels of viral mRNA were classified as active HPV infections.

In 3 HPV DNA-positive cases, RNA was not available for analysis. In these cases, p16^INK4A^ immunohistochemistry was performed. Due to strong p16^INK4A^ staining in 2 cases, these were classified as active HPV infections. Collectively, 16/18 cases with HPV DNA were finally classified as active HPV infections.

### Immunohistochemistry for SLPI

#### SLPI correlation with HPV DNA status

In 25.9% of 54 cases, there was no SLPI staining detectable. Irrespective of the HPV status, absent, weak, moderate/strong staining was detected in 38.8, 24.1 and 11.1% of SLPI-positive cases, respectively (Table III). The cases with absent/weak and the cases with moderate/strong SLPI staining were combined for further correlation analysis: out of 19 cases with moderate/strong SLPI reactivity, 89.5% were HPV-negative and 10.5% were HPV-positive; 54.3% cases with absent/weak SLPI expression (n=35) were HPV-negative, whereas 45.7% were HPV-positive. Thus, these data suggest that low SLPI expression is associated with HPV infection, whereas elevated SLPI expression appears to prevent such an infection. In the case of classifying the 3 cases with HPV RNA but no HPV DNA as active HPV infections, this correlation would be even stronger, since 2 of these cases showed absent/weak SLPI expression.

#### Lymph node-(N) status depending on SLPI expression, HPV infection status, alone and in combination

The distribution of the N0 vs. the N1–3 cases among the group of absent/weak and moderate/strong was 20 vs. 80%, and 63.2 vs. 36.8% (P=0.003), respectively. Elevated SLPI was associated with lower metastasis whereas absent/weak abundance of SLPI was associated with increased tumor burden of the neck. The distribution of patients with (n=22) and without (n=16) metastasis among the HPV-negative cases was similar. However, out of the HPV-positive cases (n=16), 13 developed metastases. The correlation between the HPV status, SLPI expression and the N-status was highly significant (P=0.003), despite the relatively small number of cases in each group (Table IV). Independent of HPV status, reduced SLPI levels were associated with tumor masses in the neck. However, the association between higher SLPI and lower prevalence of metastasis was only found in the absence of an HPV infection.

#### SLPI expression in clinically normal mucosa

SLPI immunohistochemistry of clinically normal mucosa obtained from 7 non-HNSCC patients with history of tobacco smoking showed absent/weak and moderate/strong staining in 42.9 and 57.1% cases, respectively. Patients without a history of tobacco smoking (n=12) showed absent/weak and moderate/strong staining patterns in 83.3 and 16.7% cases each (Table II). Although the absolute differences did not reach statistical significance (P=0.085) due to the small sample size the trend indicates a strong correlation between smoking habits and SLPI expression (P<0.0001).

## Discussion

To our knowledge, this is the first report showing a correlation between: i) SLPI expression and HPV infection; ii) SLPI expression, HPV infection and lymph nodal disease in head and neck cancer; and iii) SLPI expression and tobacco consumption habits in clinically normal mucosa of non-HNSCC patients.

The data presented here demonstrate that high SLPI expression is correlated with reduced prevalence of HPV infection in the investigated tumor specimens of HNSCC, while the HPV-positive cases predominate in the group of absent to low SLPI expression. Therefore, even in the presence of HPV DNA, SLPI expression may prevent an HPV infection of the upper aerodigestive tract. These findings are in agreement with the current available literature regarding the role of SLPI in oral HIV infections. For example, McNeely *et al*(24) suggested that SLPI inhibits HIV infections by blocking HIV binding to the host cells. In agreement with the effects of SLPI on HIV, Woodham *et al* recently showed that SLPI incubation of epithelial cells reduces HPV entry into these cells in a dose-dependent manner. Furthermore, SLPI was shown to bind to the Annexin A2 heterotetramer (A2t). In turn, A2t is associated with HPV insertion into and infection of epithelial cells (25). Therefore, our data support the theory of the existence of a cellular receptor for HPV and the role of SLPI as a competing agent for such receptor binding sites.

In addition, Wahl *et al*(26) suggested that SLPI mediates its anti-viral activity by affecting the host cells rather than the virus itself. In line with these findings, we found only 2 cases with high SLPI expression that were also HPV-positive. However, it remains unclear why these 2 cases had a higher lymph nodal status, suggesting that in cases with HPV infection, SLPI might lose its protective role against metastasis. Such a protective role of SLPI has previously been described (27,28), and has been assigned to the theory that SLPI affects the invasive activity of cancer cells by inhibiting enzymes promoting cancer invasion and progression. It is postulated that the absence or repression of the SLPI-antileukoprotease function, as we described here in metastatic tumor specimens, promotes spreading of the tumor by enabling degradation of surrounding tissues by proteases, secreted from the tumors.

We previously reported that significant elevation of SLPI is associated with non-metastasized HNSCC (1), a phenomenon which could be confirmed on mRNA as well as protein levels. Similarly, high statistical significance was again obtained when stratifying absent/weak and moderate/strong SLPI expression against tumor burden of the neck, using the data presented here. In contrast to our own previously published data on SLPI in metastasized and non-metastasized HNSCC showing no correlation between the expression of SLPI and the degree of tumor differentiation (1), other researchers (29,30) demonstrated such a correlation in human epidermal tumors, leading to the hypothesis that SLPI protein levels represent a negative surrogate marker for tumor progression, thus corroborating our present data regarding SLPI expression and metastasis in HNSCC. However, the exact mode of action of SLPI in carcinogenesis and metastasis remains unclear.

The role of SLPI as a potent inhibitor of neutrophil elastase appears to be an important factor resulting in the protection of the mucosa and skin against proteolysis. In addition, Chan *et al*(18) showed that total neutrophil elastase concentration and activity as well as SLPI activity was increased following exposure to cigarette smoke. This finding led us to investigate the role of SLPI in healthy mucosa of non-HNSCC patients and to correlate SLPI expression with smoking habits. In accordance with the data obtained in a rat model (18), we indeed showed that moderate/strong SLPI expression was mostly found in patients with smoking habits. In HNSCC, it is well documented that HPV-driven carcinogenesis occurs in younger patients when compared to patients with alcohol/tobacco-driven carcinogenesis (4,17). According to our data, it can be assumed that smoking-induced SLPI expression prevents HPV infection, resulting in delayed agent-dependent cancer with poorer prognosis.

In conclusion, in the present study we identified for the first time a statistically significant inverse correlation between HPV infection and SLPI expression levels, suggesting that reduced expression of SLPI facilitates HPV infection. The previously described correlation between SLPI reduction, HPV infection, and metastasis was confirmed. In addition, we provided preliminary evidence that tobacco smoking is associated with SLPI expression.

## Figures and Tables

**Table I:** Demographic and clinical characteristics of the patients and results of the tumors investigated.

	Age/gender (years)	Tumor site	TNM	HPV DNA^a^	E6*I mRNA^b^	Viral activity^c^	SLPI^d^	Smoking habits^e^
1	47/M	Lingual T	T4N2bM0		−		+++	>40 py
2	67/F	Palatine T	T2N0M0	HPV16	+	Active	+	never
3	61/M	Lingual T	T3N2bM0	HPV16		Active^f^	++	na
4	61/M	Palatine T	T4N2bM0		−		−	>40 py
5	63/M	Palatine T	T1N2bM0	HPV16	+	Active	−	na
6	53/F	Palatine T	T2N1M0	HPV16	+	Active	+++	>20 py
7	64/M	Palatine T	T1N1M0	HPV16	+	Active	−	>20 py
8	70/F	Palatine T	T3N1M0	HPV16	+	Active	+	never
9	60/M	Lingual T	T3N2bM0	HPV16	+	Active	−	na
10	51/M	Lingual T	T2N1M0	HPV16	+	Active	+	>20 py
11	76/M	Lingual T	T4N0M0		−		++	>20 py
12	53/M	Palatine T	T1N2aM0				++	<20 py
13	52/M	Palatine T	T1N2bM0	HPV16	+	Active	+	>20 py
14	54/M	Palatine T	T3N0M0		−		++	>40 py
15	59/M	Palatine T	T2N0M0		+		+	>40 py
16	47/M	Palatine T	T4N2bM0		−		+	>20 py
17	67/F	Palatine T	T3N2cM0	HPV16	+	Active	−	never
18	72/M	Lingual T	T4N2bM0		−		+	na
19	63/M	Palatine T	T4N2cM0	HPV18		Active^f^	−	>40 py
20	68/M	Palatine T	T2N0M0	HPV16/18	+	Active	+	>20 py
21	54/M	Larynx	T3N0M0				+++	>40 py
22	53/F	Larynx	T4N0M1		−		−	>20 py
23	72/M	Larynx	T3N0M0	HPV16	+	Active	−	>20 py
24	52/F	Larynx	T2N2cM0	HPV16	+	Active	−	na
25	69/M	Larynx	T2N0M0		−		++	na
26	70/M	Larynx	T4aN1M0	HPV16		Inactive^f^	+	>20 py
27	61/M	Larynx	T4N2bM0				−	>40 py
28	55/M	Larynx	T3N2cM0		−		+	>20 py
29	73/F	Larynx	T3N2cM0		+		+	never
30	48/F	Larynx	T3N0M0		+		++	>20 py
31	58/M	Larynx	T3N2cM0		−		++	>40 py
32	73/M	Larynx	T3N0M0		−		−	>40 py
33	54/F	Larynx	T4N2cM0	HPV16	−	Inactive	−	na
34	75/M	Larynx	T4aN0M0				+++	>20 py
35	54/M	Larynx	T3N1M0				+	<20 py
36	55/F	Larynx	T3N0M0				++	>40 py
37	58/M	Larynx	T4N1M0				+	<20 py
38	56/M	Larynx	T3N0M0		−		+++	>40 py
39	69/M	Larynx	T1N2cM1		−		+	>40 py
40	73/M	Larynx	T3N0M0				++	>40 py
41	58/F	Larynx	T4N2cM0				+	>20 py
42	64/M	Larynx	T3N3M0				+	>20 py
43	56/M	Tongue	T4N3M0				++	>40 py
44	57/M	Tongue	T4aN0M0		−		++	>40 py
45	53/M	Tongue	T4N2cMx				+++	na
46	43/M	Tongue	T4N2cM0		−		+	>40 py
47	57/M	Tongue	T3N2bM0	HPV16	+	Active	−	never
48	58/M	Tongue	T2N1M0	HPV16	+	Active	+	>40 py
49	52/M	Tongue	T2N0M0		−		++	>40 py
50	44/M	Tongue	T2N0M0				++	>20 py
51	64/M	Soft palate	T3N2cM1		−		−	>20 py
52	53/F	Soft palate	T1N2bM0				+	>20 py
53	71/M	Soft palate	T3N2bM0		−		+	>40 py
54	52/M	Soft palate	T2N0M0		−		+	>40 py

M, male; F, female; T, tonsil; na, data not available; SLPI, secretory leukocyte protease inhibitor.

a For final determination of HPV DNA status and genotyping, results of MPG Luminex-, LCD chip-HPV DNA detection and results of E6*I mRNA analysis were considered. Samples were defined as HPV DNA-positive when 2 of the 3 methods showed corresponding results.

b Results of E6*I mRNA analysis; when no result is given, RNA was not available for analysis (n=17).

c The HPV infection was considered biologically active when E6*I mRNA analysis and HPV DNA detection were positive for both parameters except for 3 cases^f^. For these cases, no RNA was available for analysis. However, HPV-positivity was confirmed in both DNA detection assays. In these cases, immunohistochemistry for p16^INK4A^ was carried out (data not shown) with strong staining correlating with biological activity and weak staining correlating with biological inactivity.

d SLPI antibody reactivity was scored on a semi-quantitative scale, according to Cordes *et al*(1). The correlation between SLPI and HPV status is statistically significant (P=0.005).

e Tobacco smoking habits in packs per year (py).

**Table II:** Demographic and clinical characteristics of the patients and results of the clinically normal mucosa tissue investigated for SLPI expression.

	Age/gender (years)	Mucosal site^a^	Diagnosis^b^	Smoking habits^c^	Alcohol^d^	SLPI^e^
1	51/M	Soft palate	Snoring	Quit (1.5 y)	-	+
2	63/M	Hypopharynx	Zenker’s diverticulum	Quit (1.5 y), 5 py	<10 g	++
3	51/M	Oral cavity	Branchial cyst	30 py	<10 g	++
4	52/M	Larynx	Chronic laryngitis	50 py	<10 g	−/+
5	43/F	Larynx	Reinke’s edema	40 py	<10 g	++
6	49/M	Soft palate	Snoring	30 py	<10 g	+
7	26/M	Soft palate	Chronic tonsilitis	15 py	<10 g	++
8	13/F	Soft palate	Chronic tonsillitis	-	-	+
9	6/M	Soft palate	Chronic tonsillits	-	-	+
10	53/M	Hypopharynx	Vallecula cyst	-	<10 g	+
11	59/M	Soft palate	Snoring	-	<10 g	+
12	2/F	Soft palate	Hyperplasia of tonsils	-	-	+
13	48/M	Soft palate	Snoring	-	<10 g	+
14	24/F	Soft palate	Chronic tonsillitis	-	<10 g	−
15	24/M	Soft palate	Chronic tonsillitis	-	<10 g	++
16	16/F	Soft palate	Chronic tonsillitis	-	-	+
17	46/M	Oropharynx	Chronic tonsillitis	-	-	−/+
18	22/F	Soft palate	Chronic tonsillitis	-	-	++
19	4/F	Oropharynx	Chronic tonsillitis	-	-	+

M, male; F, female; y, years; g, gram; SLPI, secretory leukocyte protease inhibitor.

a Anatomical site from where the biopsy was obtained.

b Diagnosis and reason for surgery.

c Tobacco smoke consumption in packs per year (py).

d Alcohol drinking habits of the patients. All patients reported drinking occasionally, thus are light drinkers with <10 g alcohol uptake per day.

e SLPI antibody reactivity was scored on a semi-quantitative scale, according to Cordes *et al*(1).

**Table III:** Distribution of SLPI expression levels among HPV DNA-positive and -negative cases.

	SLPI			
	
	−	+	++/+++	Total
HPV-positive	9	7	2	18
HPV-negative	5	14	17	36
Total	14	21	19	54

SLPI antibody reactivity was scored on a semi-quantitative scale, according to Cordes *et al*(1). The correlation shown here is statistically significant (P=0.005). When analyzing the SLPI negative (−)cases together with the cases with weak (+) SLPI expression against cases with moderate to strong (++/+++) expression the P=0.01. SLPI, secretory leukocyte protease inhibitor.

**Table IV:** N-status depending on HPV infection status and SLPI expression level.

	N-status		
	
	0	1–3	Total
HPV-negative and SLPI−/+	4	15	19
HPV-negative and SLPI ++/+++	12	5	17
HPV-positive and SLPI −/+	3	13	16
HPV-positive and SLPI ++/+++	0	2	2
Total	19	35	54

The correlation is statistically significant (P=0.003). SLPI, secretory leukocyte protease inhibitor.
